# Mechanisms by Which Kinesin-5 Motors Perform Their Multiple Intracellular Functions

**DOI:** 10.3390/ijms22126420

**Published:** 2021-06-15

**Authors:** Himanshu Pandey, Mary Popov, Alina Goldstein-Levitin, Larisa Gheber

**Affiliations:** Department of Chemistry and Ilse Katz Institute for Nanoscale Science and Technology, Ben-Gurion University of the Negev, P.O. Box 653, Beer-Sheva 84105, Israel; himanshu@post.bgu.ac.il (H.P.); popovma@bgu.ac.il (M.P.); alinag@post.bgu.ac.il (A.G.-L.)

**Keywords:** kinesin-5, microtubules, mitosis, cell-cycle regulation of kinesin activity

## Abstract

Bipolar kinesin-5 motor proteins perform multiple intracellular functions, mainly during mitotic cell division. Their specialized structural characteristics enable these motors to perform their essential functions by crosslinking and sliding apart antiparallel microtubules (MTs). In this review, we discuss the specialized structural features of kinesin-5 motors, and the mechanisms by which these features relate to kinesin-5 functions and motile properties. In addition, we discuss the multiple roles of the kinesin-5 motors in dividing as well as in non-dividing cells, and examine their roles in pathogenetic conditions. We describe the recently discovered bidirectional motility in fungi kinesin-5 motors, and discuss its possible physiological relevance. Finally, we also focus on the multiple mechanisms of regulation of these unique motor proteins.

## 1. Historical Outlook

**Figure 1:**
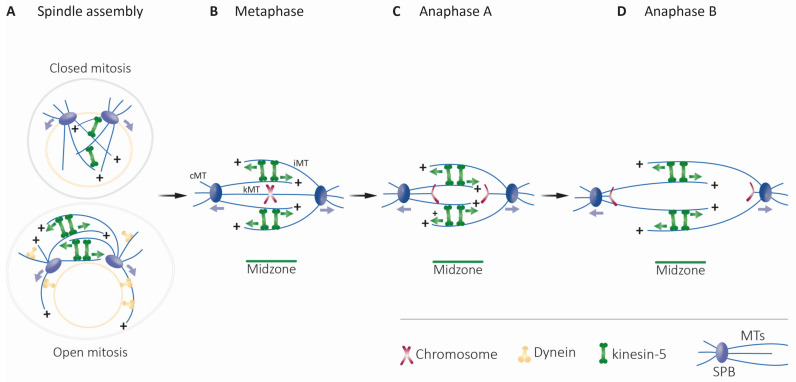
Major roles of kinesin-5 motors in mitotic spindle dynamics (adapted from [10]). (**A**) Separation of spindle poles during spindle assembly in closed (top) and open (bottom) mitosis [11]. Kinesin-5 motors mediate spindle pole-separation by crosslinking and sliding apart anti-parallel spindle MTs. Dynein, the minus-end-directed motor (yellow shape) contributes to spindle assembly in open mitosis [12,13]. Green and purple arrows indicate the direction of movement of the kinesin-5 motors and the spindle poles, respectively. (**B**) Metaphase: chromosome congression takes place at the middle of the bipolar spindle. Different types of MTs are indicated: kinetochore MTs (kMTs), astral or cytoplasmic MTs (cMTs) and interpolar MTs (iMTs). At metaphase, the chromosomes contain pairs of sister chromatids that are attached via their kinetochores to kMTs. Kinesin-5 motors stabilize the spindle by crosslinking anti-parallel iMTs of the mid-zone (indicated at the bottom). In metazoans, kinesin-5 motors maintain spindle bipolarity and drive poleward flux [14,15,16]. (**C**) Anaphase A. The onset of anaphase A. When cohesion between sister chromatids is lost, the sister chromatids are separated and migrate to the opposite spindle poles. In parallel, poleward flux-based depolymerization of kMTs takes place. (**D**) During anaphase B spindle elongation, the two spindle poles are further separated by an elongating spindle. Such elongation is mediated by cortical forces generated, for example, by dynein motors attached to the cortex which translocate along cMTs. Additional force is provided by kinesin-5-motors by sliding apart anti-parallel iMTs at the spindle mid-zone.

Following the discovery of kinesin in 1985 [1], the first evidence indicating that kinesin-related motor proteins play crucial roles in mitotic chromosome segregation emerged soon after in the early 1990s. These kinesin-related motor proteins were found to interfere with the dynamics of the mitotic spindle, a microtubule (MT)-based bipolar structure that facilitates chromosome segregation during mitosis (Figure 1). The first kinesin-5 family member that was discovered, based on its homology to the *Drosophila melanogaster* kinesin heavy chain (DmKHC, kinesin-1), was BimC from the filamentous fungus *Aspergillus nidulans*. BimC and DmKHC share 42% similarity in an N-terminal ~400 residue-long globular region now known as the motor domain, while showing very low similarity in the stalk and tail domains. Mutations in the *bimC* gene prevented spindle pole body (SPB) separation yet had no effect on organelle distribution in the cell [2]. Shortly after, two other members of the kinesin-5 family were identified by sequence homology. *Schizosaccharomyces pombe* Cut7 was found to be essential for SPB separation during spindle formation [3], while *Xenopus laevis* Eg5 (XlEg5), which is only expressed during first rapid replication following egg fertilization [4], was found to be crucial for mitotic spindle formation by moving towards the plus-end of MTs [3,5]. These three motors share higher homology relative to their homology with DmKHC [3,4] and, therefore, it was suggested that BimC, Cut7, and XlEg5 correspond to members of a separate sub-family of kinesin motors, termed the BimC sub-family [6]. The physiological roles of members of this sub-family appeared to differ from those of kinesin-1 motors. For instance, proteins of the BimC sub-family had no effect on organelle localization yet were found to be critical for centromere or SPB separation during mitotic spindle assembly [6]. Indeed, members of the BimC sub-family were identified on the basis of this function, such as *Saccharomyces cerevisiae* Cin8 and Kip1, which perform partially overlapping essential roles in spindle formation and SPB separation [7,8,9]. 

**Figure 2:**
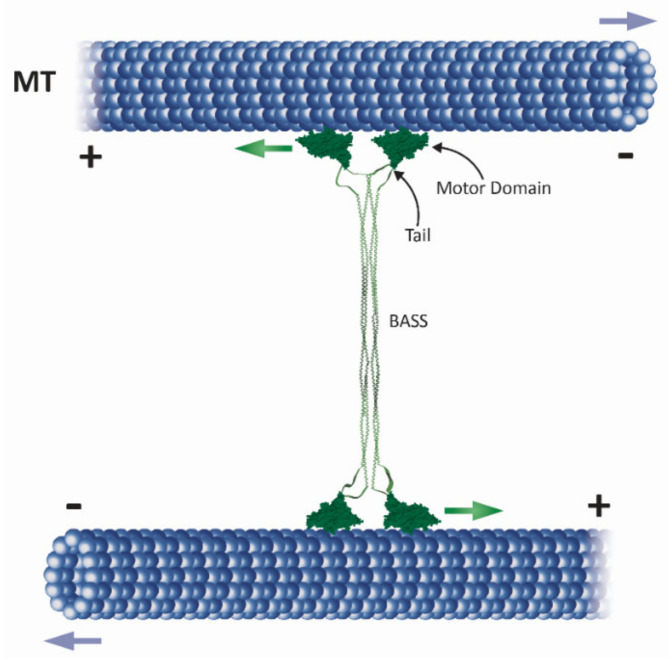
Schematic presentation of how a full-length kinesin-5 tetramer (green) crosslinks and slides apart antiparallel spindle MTs (blue). The globular motor domains and tail domains are located at the ends of the bipolar structure, connected through the central stalk which includes the bipolar-assembly (BASS) domain in the middle region [37,38]. The pair of motor domains at each end interacts with one of the two anti-parallel MTs and hence crosslinks and slides them apart. Green arrows indicate the plus-end direction of kinesin-5 motor domain movement on the MTs, while purple arrows indicate the direction of MT movement during anti-parallel sliding, with minus ends leading. Within the spindle, MT minus ends are concentrated near the spindle poles. Thus, kinesin-5 induced antiparallel MT sliding separates the spindle poles apart.

Later, other kinesin-5 motors were discovered using antibodies specific for kinesin-related proteins (KRPs), such as *D. melanogaster* Klp61F, which was shown to be a homotetrameric ~130 kDa protein essential for mitosis [17] and human Eg5 (HsEg5), whose localization is regulated by p34^CDC2^-mediated phosphorylation [18]. As the kinesin family grew, individual members were assigned to different sub-families based on function and sequence, including the BimC sub-family, now referred to as the kinesin-5 sub-family [19]. Kinesin-5 motors also share a homotetrameric structure that consists of four identical subunits that co-assemble with two pairs of catalytic motor domains on each side of a rod-shaped stalk (Figure 2) [20,21,22]. XlEg5, HsEg5, and Klp61F were shown to move towards the plus-ends of MTs in vitro [5,20,23]. In addition, it was shown that motile properties of kinesin-5 motors are important for spindle formation since a mutant of XlEg5 with reduced motile activity resulted in slower spindle assembly [24]. Interestingly, the small molecule monastrol was found to specifically inhibit XlEg5 motility in vitro, which resulted in inhibition of bipolar spindle formation and mitotic arrest [23,25,26]. This discovery prompted development of new kinesin-5-specific inhibitors for use in anti-cancer treatments [27,28,29,30]. The first evidence that kinesin-5 motors are able to crosslink MTs was demonstrated by immune electron microscopy, when *D. melanogaster* Klp61F was detected between anti-parallel spindle MTs [31]. Later it was demonstrated that in vitro, XlEg5 can walk towards the plus-ends of the two MTs it crosslinks (Figure 2) [32]. For twenty years, it was believed that kinesin-5 motors are strictly plus-end-directed, but surprisingly, three kinesin-5 motors, namely *S. cerevisiae* Cin8 and Kip1 and *S. pombe* Cut7, were shown to be bidirectional in vitro [33,34,35,36]. The fact that three kinesin-5 motors from fungi cells can switch directionality suggests that such bidirectional motility is of physiological importance.

## 2. Structural Features

Kinesin-5 motors are structurally adapted to mediate anti-parallel MT sliding, thus serving their essential function of separating the spindle poles. These motors are unique in that they act as homotetramers, with pairs of catalytic motor domains located on opposite sides of a 60 nm-long rod-like minifilament [21,22,37,38] (Figure 2). Kinesin-5 motor domains share high sequence homology to other members of the kinesin superfamily and contain ATP- and MT-binding sites [39,40,41,42,43,44]. As discussed below, the catalytic domains contain kinesin-5-specific regions and are followed by a flexible 14–18 amino acid-long neck linker that contains family-specific features and which is believed to interact directly with the catalytic domain and influence processivity and directionality. In addition, the non-motor N-terminal region is considerably longer in kinesin-5 than in kinesin-1 motors, while the unique loop 5 within the catalytic domains presents structural features specific to the kinesin-5 family that are likely responsible for the unique mechanistic properties of these proteins.

### 2.1. The Homotetrameric Kinesin-5 Complex

**Figure 3:**
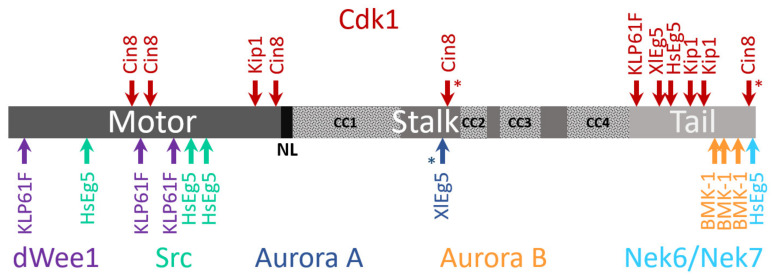
Schematic presentation of phosphorylation sites within kinesin-5 motor sequences. Motor domain, neck linker (NL), the stalk containing putative coiled-coil (CC) regions (dotted patterns), and the tail are indicated by shades of gray. Asterisks indicate an unidentified function for the phosphorylation site. For the different phosphorylation sites, the corresponding kinases (color coded) are indicated at the top (Cdk1) or bottom (other kinases). Location of the different phosphorylation sites within the amino-acid sequence is scaled according to the length of a particular kinesin-5 motor protein. Top: Phosphorylation sites for Cdk1 [17,18,31,56,57,58,61,62,63,64,65,66]; Bottom: phosphorylation sites for dWee1 [67], Src [68], Aurora A [69], Aurora B [64,70], and Nek6/Nek7 [71,72,73].

The homotetrameric bipolar structure of kinesin-5 motors is believed to be essential for the crosslinking and sliding apart of anti-parallel MTs during mitosis [32,45] since monomeric and dimeric kinesin-5 variants that do not form tetramers in vivo are non-functional [22,46]. It has also been demonstrated that the tetrameric structure is not sufficient for kinesin-5 mitotic functions in spindle dynamics, as tetrameric kinesin-5 chimeras containing the catalytic domains of kinesin-1 or chromokinesin exhibited MT-crosslinking activity but were non-functional in spindle dynamics [47]. The kinesin-5 stalk contains four regions of heptad repeat sequences that form an α-helical coiled-coil, responsible for their multimerization. Deletion studies on the *S. cerevisiae* kinesin-5 Cin8 and comparisons with other kinesin-5 proteins suggest that the coiled-coil region located immediately after the neck region (coil 1, 100–200 amino acids; Figure 3) is essential for self-interaction and sufficient for dimer Cin8 formation [46]. The central bipolar assembly (BASS, Figure 2) domain that spans ~200 residues in the central part of the stalk is essential for kinesin-5 activity and cell viability [46,48], indicating the importance of this domain in the organization of the bipolar homotetrameric kinesin-5 complex. In recent studies, the crystal structure of the *D. melanogaster* kinesin-5 Klp61F BASS domain revealed that the BASS domain consists of two anti-parallel coiled-coils at its center, stabilized by alternating hydrophobic and ionic four-helical interfaces [38]. The helixes emerge from the central part of the domain towards the N-terminal, where they bend, swap partners, and form parallel coiled-coils offset by 90°. Based on this structure, it has been proposed that the central BASS domain plays a role in transmitting forces between motors situated at the opposite ends of the molecule [38,49].

### 2.2. The C-Terminal Tail Domain

The tail domain of kinesin-1 has been reported to inhibit motility [50,51], apparently via a mechanism involving crosslinking of the two catalytic domains by the tail in the active kinesin dimer [52]. Direct evidence for an interaction between the motor and tail domains in kinesin-5 motors has recently been provided, through cryo-electron microscopy (CryoEM), for the *D. melanogaster* kinesin-5 Klp61F [53]. It has been further demonstrated that, for the human homolog HsEg5, the tails stabilize motor domains in the microtubule-bound state by slowing ATP-binding, resulting in high-force production at both homotetramer ends [53]. The tail domains of kinesin-5 motors from *X. laevis* and *D. melanogaster* have also been shown to be involved in MT crosslinking [45,54]. The tail domain of *S. cerevisiae* Cin8 was also found to be essential for the MT crosslinking in vitro [55]. However, the tail domain alone was found to be insufficient for the intracellular functions of this protein [46]. Finally, kinesin-5 homologs carry an important Cdk1 (p34/Cdc2) kinase phosphorylation site in the tail domain (Figure 3). In higher eukaryotes, this site is located within a conserved “BimC box” that is reportedly phosphorylated during mitosis [18] and see below. Phospho-deficient mutants of human, *D. melanogaster*, and *X. laevis* kinesin-5 homologs in which this site is perturbed did not localize to the spindle apparatus [18,56,57]. In fungal cells, the role of Cdk1 phosphorylation in the tail is unclear since mutations of *S. cerevisiae* and *S. pombe* kinesin-5 homologs at this site produced no obvious phenotype [58,59,60].

The influence of the tail domain on kinesin-5 directionality has been addressed in several studies. In the case of the *S. cerevisiae* kinesin-5 Cin8, which is minus-end-directed in single molecule assays under high ionic strength conditions [33], directionality preference was abolished in truncated Cin8 lacking the tail domain [55]. The motility of single tail-less Cin8 molecules was slow, processive, and bidirectional, in striking contrast to the behavior of the wild type protein. Moreover, tail-less Cin8 was unable to properly cross-link MTs in vitro or support viability of the cell when present as the sole kinesin-5 motor [55]. On the other hand, deletion of the tail domain of *S. pombe* Cut7 did not affect minus-end directionality in a multi-motor MT gliding assay, although single molecule motility of the tail-less Cut7 variant was not examined [36]. Since in the tetrameric complex the C-terminal tail domains of kinesin-5 motors are located near the N-terminal motor domains [37,38] (Figure 2) and since it has been recently demonstrated that the C-terminal tail modulates the ATPase rate of the human kinesin-5 [53], it is tempting to speculate that specific interactions between the tails and motor domains regulate the directionality of bidirectional kinesin-5 motors.

### 2.3. Neck Linker, Neck Cover Bundle and the N-Terminal Non-Motor Extension

The flexible 14–18 amino acid-long neck linker that immediately follows the C-terminus of the catalytic domain was shown to undergo ATP- and microtubule-dependent docking onto the catalytic domain. Such docking is believed to coordinate the hand-over-hand movement of the two motor domains in the kinesin dimer and control the directional motility and processivity of kinesin motors [74,75,76,77,78,79]. Studies on monodirectional kinesin motors have shown that interactions between the neck linker and the catalytic motor domain are critical directionality determinants [75,76,80,81,82,83,84,85]. Plus-end stepping directionality was maintained by chimeric kinesin motors having a catalytic motor domain from a minus-end directed kinesin-14 and neck regions from a plus-end directed kinesin-1. In addition, chimeric kinesin motors with the catalytic motor domain from plus-end directed kinesin-1 and neck regions from minus-end directed kinesin-14 persisted with minus-end stepping directionality, suggesting that regions outside the catalytic motor domain dictate directionality [76,84,86].

The kinesin-5 neck linker is longer than that of kinesin-1, a trait that was suggested to contribute to the relatively reduced processivity of kinesin-5 motors [81,87]. In contrast to kinesin-1, where prior to ATP binding the neck linker is disordered [77,88,89], the kinesin-5 neck linker is ordered and points to the minus-end of the MTs [79,90,91,92,93]. These differences may account for the different kinetics of catalytic cycle stages reported for kinesin-1 and kinesin-5 motors [39,40,42].

Recent studies have indicated that additional interactions stabilize the motor domain docking of the neck linker. Molecular dynamics simulations identified a nine residue-long N-terminal region in kinesin-1 motors (termed the neck linker cover strand (CS) or β0) responsible for a conformational change of the neck linker that is essential for force generation [94,95]. Upon ATP binding, this region contributes to the formation of a β-sheet with the neck linker, the structure that is believed to contribute to stabilization of the motor domain-docked conformation of the neck linker, which is important for plus-end-directed motility [94,95,96,97]. The N-terminal non-motor regions of kinesin-5 motors contain longer extensions, as compared to kinesin-1 motors [10,40]. However, Cryo EM and kinetic experiments indicated that the longer N-terminal region of the plus-end-directed kinesin-5 Eg5 is docked onto the motor domain with the neck linker under several nucleotide-based conditions [79,93], suggesting that although their lengths are different, the neck linker CS performs similar functions in terms of stabilizing docked conformations of the neck linker in kinesin-1 and kinesin-5 motors. Moreover, it was recently reported that the neck linker of the plus-end-directed kinesin-5 Eg5 assumes different conformations, as compared to kinesin-1, in some nucleotide-bound states [98]. Stabilization of these conformations in the Eg5 motor may rely on sequences in the neck linker and CS specific to kinesin-5 motors. The bidirectional kinesin-5 motors contain longer and divergent non-motor N-terminal extensions, as compared to kinesin-1 and the plus-end-directed kinesin-5 motors [10]. Formation of a CS bundle in the MT- and ATP-like bound state had recently been shown for a monomeric construct of the bidirectional *S. pombe* kinesin-5 Cut7 containing six N-terminal non-motor residues [99]. It is, however, possible that the additional N-terminal sequences present in the N-terminus of the bidirectional kinesin-5 motors, including Cut7, stabilize the conformations of the neck linker in a manner compatible with bidirectional motility.

### 2.4. Loop 5

In all kinesin motors, the alpha 2 helix breaks into a short loop, termed loop 5, which is located near the nucleotide-binding pocket. The sequence and length of loop 5 varies between different kinesin families, ranging from 7–10 residues in kinesin-1 to ~18 residues in kinesin-5 motors [100]. The conformation of the kinesin-5 loop 5 changes between “open” and “closed” conformations, affecting the ATPase cycle and MT affinity of kinesin-5 motors and mediating allosteric communication with the nucleotide- and MT-binding sites [92,100,101,102]. Coordinated conformational changes in loop 5, the nucleotide-binding site, and the neck linker during the ATPase cycle have been observed in solution [92,103] and were abolished upon deletion of a portion of loop 5 [92]. Specific point mutations affecting proline residues within the loop decreased both MT and nucleotide affinity and slowed the structural loop 5-dependent rearrangements that control neck linker docking [100]. In addition, deletion of loop 5 decreased the rate of MT-stimulated ADP release by kinesin-5 monomers and dimers [101]. Finally, loop 5 could synchronize the actions of kinesin-5 dimers, thus enabling initiation of dimer stepping from a two-head MT-bound state, thought to be unique to kinesin-5 motors [101,104]. Recently obtained CryoEM data for MT-bound kinesin-5 in different nucleotide-bound states support the role of loop 5 as a central coordinator of intramolecular rearrangements during the catalytical ATPase cycle [79,93].

Loop 5 is also the binding site for small molecule inhibitors specific to vertebrate kinesin-5 motors [105] that allosterically inhibit ATPase activity and directional kinesin-5 motility [23,106,107], as well as its intracellular functions [25,108,109]. Because of the clinical importance of loop 5 in binding specific kinesin-5 inhibitors, extensive mutagenesis studies have been performed with the aim of understanding the mechanism of inhibition [110,111,112,113]. The majority of mutants examined maintained activity, albeit with variably reduced sensitivity to the inhibitors. The set of mutants examined further demonstrated the inhibitor binding energy to be distributed around the pocket and that the binding pocket is able to accommodate different types of inhibitory molecules [110]. The extensive mutagenesis studies and structural data suggest a mechanism whereby allosteric inhibitors bind to a specific conformation of loop 5 and by stabilizing this conformation prevent any subsequent rearrangement of the motor domain required for the catalytical cycle. Loop 5 of the *D. melanogaster* kinesin-5 Klp61F, which is insensitive to allosteric inhibitors such as monastrol [112], assumes a different conformation than do the vertebrate kinesin-5 motors when bound to MTs in the presence of AMP-PNP [79,114]. This supports the notion that kinesin-5 allosteric inhibitor binding is dependent on a vertebrate-specific conformation of loop 5.

### 2.5. Loop 8

The flexible loop 8 breaks the beta strand 5 of the kinesin motor domain into two sections [115]. In the MT-bound state of the motor, loop 8 faces the MT lattice and thus is thought to be part of the MT-binding domain [116,117]. It has also been shown that in the dimeric structure of kinesin-1, loop 8 of one motor domain interacts with loop 10 of the other. This interaction was proposed to serve as an inter-subunit connection, allowing communication between the motor domains in an active dimer [116]. The amino acid sequence of *S. cerevisiae* Cin8 includes a large (99 amino acid) insertion in loop 8, which is not essential for intracellular function [7]. Loop 8 of Cin8 was found to regulate the directionality of the motor, since replacement of this insert with the short loop 8 of the *S. cerevisiae* Kip1 induced bias to the minus end-directed movement of Cin8 in a single-molecule motility assay [33]. It had been suggested that loop 8 can regulate the directionality of Cin8 through its noncanonical binding to MTs [118]. However, it was recently demonstrated that, in the monomeric form of Cin8, loop 8 had no effect on the directionality of Cin8 in a multi-motor MT gliding assay [119]. In contrast, a study from our laboratory demonstrates that loop 8 of Cin8 directly interacts with the MTs and induces bidirectional motility in both full-length and dimeric forms of Cin8 (Himanshu et al., in preparation). These results may indicate that in order for loop 8 to regulate the directionality of Cin8, two motor domains have to interact with the same MT, as in the scenario of native tetrameric and dimeric forms of Cin8.

## 3. Motor Activity

Kinesin-5 motors perform essential functions in spindle dynamics by binding to and moving along MTs. Because of their bipolar structure, these motors can crosslink and slide apart anti-parallel spindle MTs (Figure 1 and Figure 2), thus providing the outwardly-directed force that separates the spindle poles.

### 3.1. Velocity, Processivity and Anti-Parallel MT Sliding

Since their initial discovery, the mechano-chemical cycle of kinesin-5 motors has been extensively studied using monomeric, dimeric, and full-length variants [39,90,93,100,102,106,120]. Such studies revealed that the MT-stimulated ATPase rate of monomeric kinesin-5 is slower than that of kinesin-1 (~7/s vs. ~50/s) [90,120,121], with Pi release being the rate-limiting step [106]. Kinesin-5 motors, moreover, exhibit unique motile properties [39,40,42,122,123], as seen below. Since kinesin-5 motors carry their catalytic domain at the N-terminus, they were initially thought to be exclusively plus-end-directed. Indeed, in multi-motor MT gliding assays, where in vitro-polymerized MTs are translocated by surface-bound motors, monomeric and dimeric human, mouse, and *Xenopus laevis* kinesin-5 variants were shown to move MTs with the minus-ends leading, consistent with a plus-end directionality of the motors [81,124,125,126]. In contrast to kinesin-1, the velocity of MT gliding driven by kinesin-5 motors was similar for monomers and dimers, suggesting that in order to translocate MTs, coordination between the two motor domains within a dimer is not essential, such that single motor domains can mediate motility so long as the MT is maintained near the surface via the binding of neighboring motors [124,126]. Plus-end-directed MT gliding of full-length kinesin-5 proteins from human, *D. melanogaster*, *X. laevis*, and *S. cerevisiae* was also demonstrated [20,32,34,35,56,81,127,128]. However, the velocity of kinesin-5-mediated MT gliding was considerably slower (10–70 nm/s) [39,123] than that of kinesin-1 motors (~500–1000 nm/s) [1,129,130,131], indicating differences in the mechanochemical cycle [42]. Finally, in single molecule fluorescence motility assays, kinesin-5 motors from vertebrates were also shown to be slow and plus-end-directed [23,81,87,128,132], although the velocity and directionality of single molecules from other members of the kinesin-5 sub-family differed significantly (see below).

Processivity, defined as the number of consecutive steps taken by a motor before detaching from MT tracks, varies among the different kinesin sub-families [39]. Although kinesin-5 motors were shown to be processive, such processivity is lower than that of kinesin-1 motors. Dimeric human kinesin-5 variant takes ~8 steps on average before detaching [133], while the dimeric kinesin-1 motors take several hundreds of steps [134]. Kinesin-5 processivity was shown to be force-dependent in a bead-based optical trap assay [133,135]. The processivity of full-length kinesin-5 motors is higher as compared to that of dimeric constructs, yet is still lower than that of kinesin-1 motors [23]. Finally, it was suggested that the longer neck-linker region (see below) is associated with the reduced processivity of kinesin-5 motors [81,87]. The low processivity of kinesin-5 motor could reflect adaptation to their intracellular function, where ensembles of kinesin-5 motors crosslink interpolar spindle MTs (see below), reducing the requirement for high processivity.

MT architecture within the mitotic spindle is such that the plus-ends are pointing towards the midzone (Figure 1). Therefore, to separate the spindle poles, the bipolar kinesin-5 motors must crosslink and slide anti-parallel interpolar MTs of the midzone in a plus-end-directed manner. Indeed, crosslinking and anti-parallel sliding of MTs was demonstrated for kinesin-5 homologs from yeast to vertebrates [33,34,45,127,128]. *X. laevis* XlEg5 was shown to walk in a plus-end-directed manner along the two MTs it crosslinks [32], with force production during anti-parallel sliding being correlated with the length of the zone of MT overlap [136]. Under physiological salt conditions, crosslinking two MTs was shown to activate *X. laevis* Eg5. On a single MT, this motor exhibited diffusive bidirectional motility, which may be attributed to an additional MT-binding domain in the Eg5 tail [54]. This diffusive mode switches to plus-end-directed processive motility upon the binding of a second MT to produce anti-parallel MT sliding [128]. These findings demonstrate the adaptation of kinesin-5 motors to perform anti-parallel sliding only when bound between two anti-parallel MTs.

Mitotic spindle motors function in a crowded environment, in the presence of other MT-binding motor and non-motor proteins. Several studies have addressed the functionality of kinesin-5 motors in the presence of other motors. *X. laevis* Eg5 was shown to antagonize and slow MT motility driven by the fast kinesin-1 in gliding assays [137], while the *D. melanogaster* kinesin-5 Klp61F was shown to antagonize the minus-end-directed kinesin-14 Ncd [48]. These findings may correspond to the “braking” activity of kinesin-5 motors within the spindle [136,138,139,140] and reflect their ability to balance spindle forces during mitosis.

### 3.2. Bidirectional Motility of Fungal Kinesin-5 Motors

Although the plus-end-directed motility of kinesin-5 motors is essential for separating spindle poles during spindle assembly and anaphase B, two earlier studies reported that *S. cerevisiae* kinesin-5 Cin8 is minus-end-directed when moving as a single molecule in high ionic strength conditions, and switches directionality in several other experimental conditions [33,34]. Following the first reports on Cin8 bidirectionality, the kinesin-5 homologs *S. cerevisiae* Kip1, and *S. pombe* Cut7 were also reported to be bidirectional and exhibit switchable directionality under certain in vitro experimental conditions [35,36]. Subsequent work revealed intramolecular domains that affect the directionality of these three kinesin-5 motors [10], such as the N-terminal non-motor extension [141,142], the C-terminal tail domain [55], and the large insert in the loop 8 within the catalytic domain [33], which was also demonstrated to induce non-canonical binding of Cin8 to MTs [118]. Phosphorylation of three cyclin-dependent kinase 1 (Cdk1) sites in the catalytic domain of Cin8, which regulates its intracellular activity [58,60,61,143], with two of these sites being located in large loop 8, was also shown to affect Cin8 directionality [144].

Intermolecular interactions between bidirectional kinesin-5 motors or between these motors and non-motor proteins also affect motor directionality. Based on multi-motor MT gliding and anti-parallel MT-sliding assays, it was suggested that the switch from fast minus-end- to slow plus-end-directed motility results from the coupling of *S. cerevisiae* Cin8 and Kip1 motors through the MT with which they both interact [34,35,145]. This model, which was recently supported by a theoretical study [146], does not apply to *S. pombe* Cut7, which was shown to be minus-end-directed in both single molecule and multi-motor MT gliding assays [36]. It has also been demonstrated that crowding of Cut7 by motor and non-motor proteins can drive directional switching from minus- to plus-end-directed motility, with it being suggested that such crowding reflects a steric blockage mechanism [142]. Finally, it was reported that accumulation of Cin8 into clusters slowed motility and induced a switch from minus- to plus-end-directed motility [62]. This study further proposed that since the ability to move in both directions is an intrinsic property of Cin8 tetramers [33,55,147], specific intermolecular interactions between Cin8 tetramers in a cluster control the directionality of this motor [62]. Based on the characterization of single-molecule and cluster motility, a recently developed model predicts that directionality switching of the bidirectional Cin8 is caused by an asymmetric response of its active motion to opposing forces, referred to as drag [148]. The model showed excellent quantitative agreement with experimental data obtained under high and low ionic strength conditions. This recent analysis identified a robust and general mechanism that explains why bidirectional motor proteins reverse direction in response to experimental conditions, including changes in motor density and molecular crowding and in multimotor motility assays [148].

### 3.3. Interaction with MT Ends

Several lines of evidence suggest that in addition to moving along MTs, kinesin-5 motors also interact with and control the dynamics of MT ends. *S. cerevisiae* Kip1 was shown to bind to MT plus-ends in cells and to follow the plus-end of growing and shrinking MTs, which is likely related to Kip1-mediated MT stabilization at the end of anaphase B [35]. Plus-end accumulation and stabilizing activity in vitro were also demonstrated for a dimeric chimera comprising the *X. laevis* Eg5 catalytic domain and a kinesin-1 stalk [132]. Protection of MT depolymerization by this chimera was inhibited by specific kinesin-5 inhibitors [149]. On the other hand, deletion of *S. cerevisiae* Cin8 and inactivation of the *D. melanogaster* kinesin-5 Klp61F led to longer and more stable MTs [150,151,152], indicating that these motors destabilize MTs. Finally, Cin8 was shown to accumulate and track the minus-ends of dynamic MTs in vitro [62]. The physiological significance of such behavior remains to be elucidated.

## 4. Intracellular Function

### 4.1. Roles in Dividing Cells

#### 4.1.1. Structure and Dynamics of the Mitotic Spindle

In eukaryotic cells, separation of duplicated chromosomes during mitosis is mediated by the MT-based mitotic spindle [153]. MTs of the spindle are arranged with their minus-ends concentrated at the centrosomes or SPBs, and are divided into three classes based on their function [154,155] (Figure 1). The plus-ends of the kinetochore MTs (kMTs) capture chromosomes at protein structures termed kinetochores and facilitate chromosome movement within the spindle [156,157]. Cytoplasmic or astral MTs (cMTs) are captured at sites on the cell cortex and mediate spindle positioning [158,159,160]. Interpolar MTs (iMTs) overlap in an anti-parallel array in the middle region of the spindle, the midzone [161,162,163], and are focused in parallel arrays near the poles [155]. The mitotic bipolar spindle assembly is established by the separation of the two spindle poles into a bipolar structure where the two spindle poles are located at the opposite ends of the overlapping iMT array (Figure 1A) [164,165,166]. This separation is primarily accomplished by pushing forces applied from within the spindle on iMTs [7,8,25,63,167]. In higher eukaryotes that divide via open mitosis accompanied by nuclear envelope breakdown, [166,168,169,170], additional pulling forces that contribute to centrosome separation are applied by nuclear envelope- or cortex-bound dynein motors on cMTs (Figure 1A) [171,172,173,174,175,176]. Following mitotic spindle assembly, chromosomes are captured at the kinetochores by the plus-ends of MTs emanating from the spindle poles, ultimately leading to their congression to the middle of the spindle in metaphase (Figure 1B). Attachment of all chromosomes to kMTs emanating from both poles and consequent inactivation of the spindle assembly checkpoint leads to separation of the sister chromatids by degradation of the cohesion complex [177,178,179] and their movement to the opposing poles via depolymerization of the kMTs at their plus-ends (anaphase A, Figure 1C) [180,181]. Following anaphase A, the spindle elongates to further separate the two groups of chromatids (anaphase B). This event is largely realized by pushing forces from within the spindle and by polymerization of iMTs at their plus-ends (Figure 1D) [16,182].

#### 4.1.2. Bipolar Spindle Assembly, Maintenance, and Elongation

**Figure 4:**
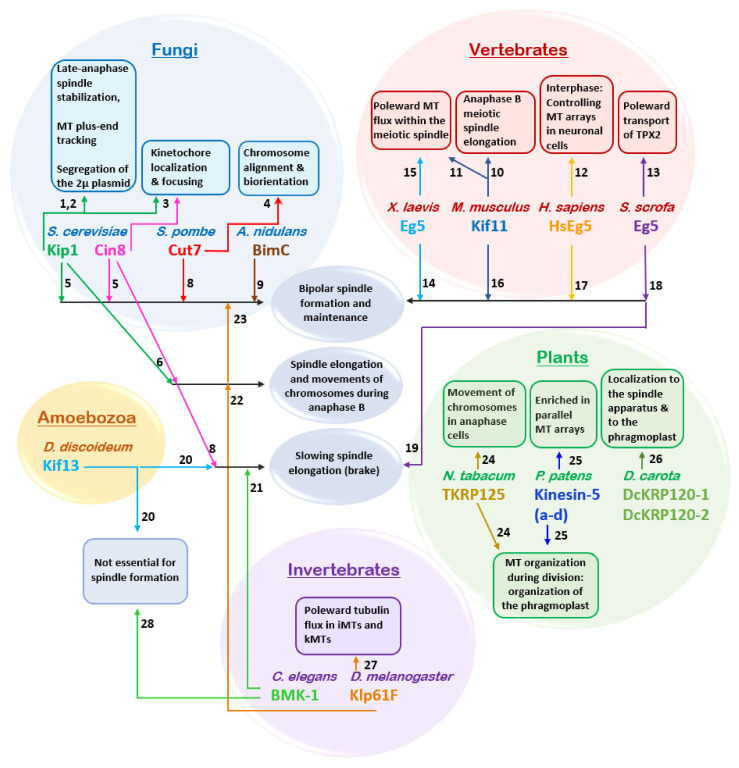
Physiological roles of kinesin-5 motors. Functions common to at least two kinesin-5 motors are indicated in blue ellipses. Functions identified for a single kinesin-5 motor are indicated in framed rectangles. Colored spheres indicate different groups of species. Arrows point to specific intracellular functions. Numbers indicate the related references: (1) [35]; (2) [196,197,198]; (3) [151,199,200,201,202]; (4) [203]; (5) [7,8,9,204]; (6) [189,190,191,192]; (7) [139]; (8) [3,205]; (9) [2]; (10) [206]; (11) [207]; (12) [208,209]; (13) [210,211]; In [211], human Eg5 was expressed in *Sus scrofa* cells. (14) [4,5,56]; (15) [212,213]; (16) [184]; (17) [18,214]; (18) [210]; (19) [195]; (20) [187]; (21) [140,215]; (22) [31,216]; (23) [17]; (24) [217]; (25) [188]; (26) [218]; (27) [14,216]; (28) [70].

Members of the kinesin-5 sub-family have been shown to perform essential roles related to mitotic spindle dynamics in eukaryotic cells (Figure 4) [15,40,42,122,123]. The key roles of kinesin-5 motors, namely the establishment and maintenance of a mitotic spindle (Figure 4), relies on their ability to crosslink and slide anti-parallel MTs apart, due their unique bipolar structure (Figure 2). These functions are highly conserved in eukaryotes, with numerous studies indicating that a loss of kinesin-5 function leads to mitosis failure due to a lack of spindle pole separation prior to spindle assembly, or as a result of spindle collapse after establishment of the bipolar spindle [5,7,17,18,26,183,184,185]. However, it remains to be fully established whether or not anti-parallel MT sliding is essential for spindle assembly, or if MT crosslinking by kinesin-5 motors is sufficient [186]. To date, only two organisms, the nematode *Caenorhabditis elegans* and the slime mold *Dictyostelium discoideum* have been reported as being able to form a functional bipolar spindle in the absence of kinesin-5 motors (Figure 4) [70,187]. The moss *Physcomitrella patens* can be partly considered a third exception as although kinesin-5 is not essential for mitotic spindle formation in this organism, it is still required for post-anaphase spindle/phragmoplast integrity and chromosome segregation (Figure 4). [188]. The role of kinesin-5 motors in anaphase B spindle elongation, however, remains controversial. An anaphase-facilitating function was demonstrated in *S. cerevisiae* [189,190,191,192] and insect cells [193] (Figure 4). Moreover, the *S. cerevisiae* kinesin-5 motors Cin8 and Kip1 were shown to be partially destabilized so as to prevent anaphase onset in cells carrying damaged DNA [194]. In other cases, it was suggested that kinesin-5 motors slow spindle elongation rates by applying “brakes” involving the crosslinking of spindle MTs [139,140,187,195]. (Figure 4). Interestingly, some features of kinesin-5 homologs are characteristic to specific taxonomic groups, such as the bidirectional motility of kinesin-5 motors in fungi, the abundance of kinesin-5 motors in plants or the poleward transport of kinesin-5 motors by TPX2 and dynein in vertebrates (Figure 4).

#### 4.1.3. Models for Maintaining Spindle Bipolarity

Biophysical models describing the role of kinesin-5 motors in maintaining the bipolar spindle structure have been proposed in different organisms, based on the force-balanced model suggested for *S. cerevisiae* spindles [204,219]. According to this model, bipolarity of the spindle is maintained by a balance of inwardly- and outwardly-directed forces exerted by the minus-end-directed kinesin-14 Kar3 and the kinesin-5 motors Cin8 and Kip1, which are plus-end-directed between anti-parallel MTs [33,34,35]. In support of this notion, it has been demonstrated that in *S. pombe*, spindles could been assembled when kinesin-5 and kinesin-14 motors were simultaneously deleted [167]. Normally, the onset of anaphase B is triggered by a shift in the balance of these forces in favor of spindle elongation. Similar mechanisms were suggested for *D. melanogaster* embryos [220] and *X. laevis* [221,222].

#### 4.1.4. Effects on MT Turnover and Dynamics

In higher eukaryotic cells, kinesin-5 motors were shown to affect the poleward turnover of tubulin (poleward flux) in kMTs and iMTs [212,213,223], which contributes to chromosome congression and separation, respectively [14,31,216]. Poleward flux is not observed in yeast cells [224,225], however, kinesin-5 motors have been shown to affect MT dynamics in *S. cerevisiae* cells [35,150,151]. In addition, kinesin-5 motors have been shown to bind to kinetochores, focus kinetochore clusters, and limit the length of kMTs in *S. cerevisiae* [151,199,200,201,202]. Finally, the *S. cerevisiae* kinesin-5 Kip1 is required for segregation of the 2-micron plasmid [196,197,198], the function of which can be related to the minus-end-directed motility of Kip1 (Figure 4) [35].

#### 4.1.5. Functions at the Spindle Poles

**Figure 5:**
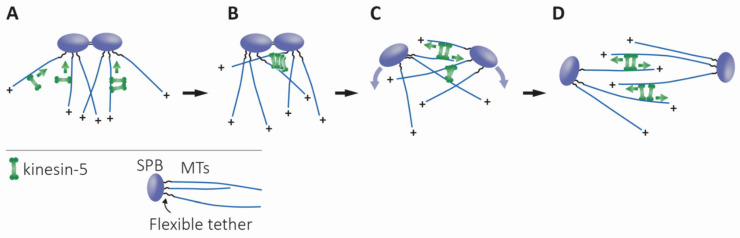
Proposed model for the physiological role of the bidirectional motility of fungi kinesin-5 motor proteins, adapted from [62]. (**A**) Prior to spindle assembly, the minus-end directed motility of bidirectional kinesin-5 motors causes their accumulation at the minus-ends of the MTs, near the SPBs. (**B**) At this stage, MTs from duplicated spindle poles come into antiparallel contact due to thermal pivoting, facilitated by flexible tether linking MT minus-ends and the SPB [235]. Kinesin-5 motors, accumulated in clusters near the SPBs, capture these antiparallel-oriented MTs, crosslink, and slide them apart via their plus-end directed motility. This plus-end directed motility is achieved by the clustering of kinesin-5 motors near the spindle poles and the binding to the two antiparallel MTs [62,148]. (**C**) The initial separation of the spindle poles, caused by the antiparallel sliding of MTs, generates additional MT overlap—which creates additional sites for the kinesin-5 motors to crosslink and slide apart antiparallel MTs. (**D**) Formation of the final bipolar spindle structure is achieved. The curved purple arrows indicate the direction of SPB movement during separation. Green arrows indicate the direction of motor motility.

Several reports suggest that localization near the spindle poles is important for kinesin-5 function. First, in a number of organisms, kinesin-5 motors were found to be enriched near centrosomes or SPBs [5,56,188,226,227,228]. Second, it had been demonstrated that in higher eukaryotes undergoing open mitosis, kinesin-5 motors are actively transported towards the poles by the dynein–dynactin complex [226,227,229]. The Ran-regulated spindle-pole localizing factor TPX2 [230] was found to recruit kinesin-5 to the poles in *Xenopus* spindles [231], possibly via connecting the kinesin-5 to the dynein complex, as demonstrated in mammalian cells [232]. Dynein-dependent poleward kinesin-5 delivery is absent in yeast that undergo closed mitosis. However, kinesin-5 motors also co-localize with spindle poles prior to spindle assembly in yeast cells [3,62]. The discovery of switchable kinesin-5 motor directionality in budding and fission yeast [33,34,35,36] suggests possible physiological roles for this ability in closed mitosis (Figure 5). Bidirectionality may be the evolutionary adaptation that allows these motors to localize to spindle poles prior to spindle assembly by minus-end directed motility on nuclear MTs, without the function of the minus-end-directed dynein [10,62]. At this location, kinesin-5 motors can capture anti-parallel MTs emanating from the neighboring pole and mediate their anti-parallel sliding by plus-end directed motility, thus promoting spindle assembly (Figure 5) [62]. In support of this hypothesis, a recent computational model identified a set of functions essential for the generation and stability of spindle bipolarity in fission yeast, showing that the bidirectionality of kinesin-5 motors enables proper localization to the spindle apparatus, which is necessary for spindle assembly [233].

Building a stable bipolar spindle requires enough antiparallel MT interactions for the separation of the poles by plus-end-directed kinesin-5 motor motility. An interesting question is whether the MTs emanating from the unseparated neighboring poles are flexible enough to be captured and aligned by the kinesin-5 motors accumulated at the poles. In recent years, a few studies in yeast have shed light on this question. It has been shown in *S. pombe* that random rotational pivoting helps microtubules from the neighboring poles to come into contact prior to spindle assembly [234]. Recently, by engineering a flexible tethering protein, Spc110, which links microtubule minus ends to the SPB core, Fong et al. demonstrated that thermal microtubule pivoting is specifically important during early spindle assembly of *S. cerevisiae*, and that this pivoting motion is sufficient to bring microtubules from neighboring poles into contact [235]. In support of this notion, in high-voltage electron tomography of early mitotic spindles in *S. cerevisiae* it has been shown that, after SPB duplication, while the SPBs are still connected by a bridge, the MTs from each SPB interdigitate at sharp angles [236]. According to our model, such overlap of MTs emanating from the neighboring poles, induced by their flexible pivoting, is sufficient for the fungi kinesin-5 motors located at the poles, to capture these MTs and mediate spindle assembly via plus-end directed antiparallel MT sliding (Figure 5).

### 4.2. Roles in Non-Dividing Cells

Although the majority of studies addressing the physiological roles of kinesin-5 motors have focused on their roles in mitosis, several studies have also revealed roles for these proteins in interphase, including regulation of the biogenesis and function of Ago1-complexes [237], as well as Golgi organization and polypeptide synthesis, in human cells [238,239]. The most extensively studied function of kinesin-5 motors in interphase is in neuronal cells, due to the clinical relevance of this system. Kinesin-5 motors are expressed in post-mitotic neurons [240], where they may play a role in the proper development of mammalian neuronal processes, including axon growth cone guidance, elongation and branching [208,209,241] and modulation of neuronal growth and migration [242]. In dendrites, inhibition of kinesin-5 motors causes changes in cell morphology and MT organization. Taken together, these observations suggest that kinesin-5 regulates axon and dendrite growth, acting as a brake on MT bundle assembly [243]. It is likely that cytoplasmic dynein is the principal motor whose forces are attenuated by the braking function of kinesin-5 [243], acting via two other kinesin motors, kinesin-6 and kinesin-12, that may also participate in co-regulation of MT patterns in axons and dendrites [240].

The research on kinesin-5 functions in plant cells is considerably less developed than in fungi and metazoans. However, several studies have revealed that kinesin-5 motors play non-canonical roles in plant cells [244]. For example, in *Nicotiana tabacum*, kinesin-5 was reported to be involved in separating anti-parallel microtubules in the phragmoplast, a MT-based structure formed during late cytokinesis (Figure 4) [217]. In *Arabidopsis thaliana*, disruption of kinesin-5 activity leads to disorganization of intracellular microtubules during interphase, as well as spindle formation [183]. Additional studies are needed to fully characterize the multiple functions of kinesin-5 motors in plant cells.

### 4.3. Phosphoregulation

In the eukaryotic cell cycle, mitotic events are coordinated via phosphorylation by specific kinases [245,246,247,248,249,250]. The balance between phosphorylation and dephosphorylation was demonstrated to govern the localization of these kinases within the mitotic spindle and regulate their functions. The amino acid sequences of kinesin-5 motors contain multiple phosphorylation sites located within and outside the motor domain (Figure 3), a large number of which have been demonstrated to regulate kinesin-5 [42,122,123].

It has been reported for a number of eukaryotes that a conserved sequence termed the “BimC box”, located in the C-terminal tail, possesses a conserved phosphorylation site for cyclin-dependent kinase 1 (Cdk1; Cdc28 in yeast) (Figure 3). For human Eg5, phosphorylation at this site regulates spindle targeting, association, and localization, with characteristic concentration of the kinase at the poles [18]. Moreover, Cdk1 is required for interaction with dynein through the p150_glued_ subunit of dynactin [63]. Consistently, *X. laevis* mutants phosphodeficient in the Eg5 BimC box showed disrupted kinase localization to the mitotic spindle [56], while in *X. laevis* spindles assembled from an egg extract, phosphorylation by Cdk1 increased the binding of Eg5 to MTs [64]. In higher plants, such as *Arabidopsis thaliana*, tobacco, and carrot, kinesin-5 motor activity in stabilization and sliding apart anti-parallel MTs is Cdk1-dependent [218,251,252,253], and is regulated by phosphorylation of the BimC box. In *D. melanogaster* embryonic mitotic spindles, Klp61F motors, phosphorylated at a Cdk1/cyclin B consensus domain within the BimC box, concentrate in the mid-zone region of interpolar MT bundles [31]. In addition, it was shown that Klp61F is phosphorylated both within the BimC box and in the motor domain [67,228]. This leads to dynamic localization of Klp61F throughout the spindle and cross-bridging between both parallel and anti-parallel MTs [31,254]. These findings hint at coordination with another important cluster of phosphorylation sites located in the N-terminal motor domain, in close vicinity to the ATP-binding pocket.

In yeast, Cdk1 also phosphorylates kinesin-5 motors although the mechanism of phosphoregulation appears to be different than employed in higher eukaryotes. In fission yeast, Cdk1 phosphorylation at the C-terminal of the kinesin-5 Cut7 is not required for association with microtubules [59]. Both *S. cerevisiae* Cin8 and Kip1 lack the BimC box motif, although Cdk1 phosphorylation consensus sequences are present in their C-terminal tails [61]. Phosphorylation in the Kip1 motor domain by Cdk1 was shown to be required for spindle pole separation [61]. Cin8 was, furthermore, demonstrated as being differentially phosphorylated during anaphase at three Cdk1 sites located in its motor domain [58]. Such phosphorylation induces Cin8 detachment from spindles and reduces the spindle elongation rate [58], with each of the three sites playing unique roles in phosphoregulation of Cin8 [60,255]. In vitro experiments with Cin8 phospho-mutants suggest that phosphorylation of the three sites within the motor domain provides fine tuning of motor activity. Accordingly, phospho-mimetic mutants possess weaker MT-motor interactions, increased motor velocity, and minus-end-directionality [144].

Aside from Cdk1, other kinases have been shown to regulate kinesin-5 motors (Figure 3). For example, the tail domain of HsEg5 contains a phosphorylation site recognized by Nek6/Nek7 kinases. This site contributes to the accumulation of Eg5 at the spindle poles and is necessary for subsequent spindle pole separation [71,72]. Phosphorylation of the HsEg5 motor domain by Src kinase tunes motor activity so as to obtain optimal spindle morphology [68]. The *D. melanogaster* kinesin-5 Klp61F is phosphorylated at the motor domain by the Wee1 tyrosine kinase, which may thus affect Loop 5 conformation and motor function [67]. Finally, regulation by the Aurora kinases was also reported for some kinesin-5 motors. *C. elegans* kinesin-5 was reported to be phosphorylated at the tail domain by the Aurora B kinase, which regulates its spindle localization [64,70]. The *X. laevis* kinesin-5 Eg5 was shown to be phosphorylated by the Aurora A kinase at the stalk domain [69], although such phosphorylation was found to be non-essential for spindle formation [64]. Work in the coming years is likely to reveal the complex mechanism by which phosphorylation by different kinases regulates kinesin-5 motor functions.

### 4.4. Other Post-Translation Modifications

It had recently been reported that the motor domain of the human kinesin-5 Eg5 undergoes acetylation at a specific lysine residue (K146). Using the acetylation-mimic K146Q mutant, it was further shown that such acetylation disrupts a salt-bridge formed between K139 and D91 in a neighboring α-helix [256]. This disruption increased the coupling of the neck linker to the catalytic domain, enhanced motor performance under load, and increased binding to MTs [256]. While this study clearly revealed that acetylation can regulate the functions of kinesin-5 motors, additional studies are needed to expand on the generality of this finding.

## 5. Kinesin-5 Motors and Pathological Conditions

Since mitosis is a fundamental process in proliferating cells, the mitotic spindle offers a prime target for cancer therapy [257,258,259]. Inhibitors that act by “shutting down” key components of kinesin-5 motors may thus cause mitotic arrest of proliferative tissues. Historically, chemotherapeutic drugs designed to interfere with the cell cycle specifically targeted microtubules. Such drugs include the clinically used taxanes and *vinca* alkaloids [260,261]. The development of resistance to microtubule-targeted drugs and severe side effects raised interest in alternative targets. The human kinesin-5 Eg5, which is involved in the formation of a bipolar spindle, meets the criteria for an appropriate target in cancer. Eg5 is over-expressed in hematological malignancies and many solid tumors, such as breast, ovarian, bladder, and pancreatic cancers, yet is found at negligible levels in non-proliferative tissues [262,263,264,265]. In breast cancer patients, Eg5 over-expression is associated with poor prognosis. Accordingly, Eg5 is considered a typical oncoprotein and has been proposed as a potential prognostic biomarker and target for therapeutic agents in oral and breast cancer treatment [266,267]. Eg5 possesses unique structural features that selectively dispose it to small molecule inhibitors. Within the Eg5 motor domain, loop 5 presents a prolonged structure, forming a druggable allosteric pocket located between helix α3 and loop 5, [91]. Moreover, loop 5 is flexible [268] and, as mentioned in the “structural information” section, undergoes an “open” to “closed” structural transition that correlates with rearrangements at the active site during the ATPase cycle [92,100,101,102]. Since the discovery of monastrol, a specific vertebrate kinesin-5 inhibitor that binds to loop 5 [25,26], a variety of Eg5-specific inhibitors have been developed [269,270,271]. These inhibitors arrest cells in mitosis, producing characteristic monoastral spindles [108,109,149], and lead to apoptosis in proliferative tissues either by binding to loop 5 or other allosteric sites and inhibiting ATPase activity [30,102,103,111,149,272]. To develop and test promising Eg5 inhibitors, different approaches are used, including in silico drug-design predictive tools that seek to bridge computational modeling with drug design and cancer therapy [259,273,274]. Based on these studies, several kinesin-5 inhibitors are currently in clinical trial [29,275,276,277].

Several reviews shed light on the state of the art in terms of kinesin-5 inhibitors serving as anti-tumor drugs, reporting the results of clinical trials of the most potent candidates and offering reasons for their poor clinical outcome [40,259,278,279,280]. To summarize, more than 40 Phase I and II clinical trials assessing inhibitors of the kinesin-5 Eg5, starting with the first generation drug ispinesib [29] and followed by filanesib [276], were suspended or discontinued. When used as monotherapy, Eg5-targeting agents have only been moderately successful, instead causing common adverse effects, such as neutropenia [278]. Therefore, a multi-drug combination is likely to improve efficacy [281]. It has also been demonstrated that the kinesin-12 motor Kif15 can functionally replace inhibited Eg5 and cause drug resistance [282]. To overcome Kif15-mediated cancer drug resistance, a strategy involving a combination drug therapy employing both inhibitors has been suggested [283].
